# Preclinical and Clinical Studies on Antioxidative, Antihypertensive and Cardioprotective Effect of Marine Proteins and Peptides—A Review

**DOI:** 10.3390/md14110211

**Published:** 2016-11-18

**Authors:** Ida-Johanne Jensen, Hanne K. Mæhre

**Affiliations:** Norwegian College of Fishery Science, Faculty of Biosciences, Fisheries and Economics, UIT The Arctic University of Norway, N-9037 Tromsø, Norway; hanne.maehre@uit.no

**Keywords:** marine, proteins, peptides, bioactive, antioxidative, clinical trials, preclinical, animal studies

## Abstract

High seafood consumption has traditionally been linked to a reduced risk of cardiovascular diseases, mainly due to the lipid lowering effects of the long chained omega 3 fatty acids. However, fish and seafood are also excellent sources of good quality proteins and emerging documentation show that, upon digestion, these proteins are sources for bioactive peptides with documented favorable physiological effects such as antioxidative, antihypertensive and other cardioprotective effects. This documentation is mainly from in vitro studies, but also animal studies are arising. Evidence from human studies evaluating the positive health effects of marine proteins and peptides are scarce. In one study, a reduction in oxidative stress after intake of cod has been documented and a few human clinical trials have been performed evaluating the effect on blood pressure. The results are, however, inconclusive. The majority of the human clinical trials performed to investigate positive health effects of marine protein and lean fish intake, has focused on blood lipids. While some studies have documented a reduction in triglycerides after intake of lean fish, others have documented no effects.

## 1. Introduction

Cardiovascular diseases (CVDs) are a group of diseases affecting the heart and blood vessels and they are the largest cause of morbidity and premature deaths worldwide [1] accounting for 31% of all global deaths in 2012 [2]. The development of CVDs is associated with several risk factors, both modifiable and non-modifiable, and the danger of developing CVD increases considerably with the number of risk factors present [3]. Gender, heredity and increasing age are risk factors that are non-modifiable. Modifiable risk factors are often life-style related and may be associated with oxidative stress. Tobacco smoking, physical inactivity, diabetes mellitus, obesity and overweight are, along with hypertension and dyslipidemia, examples of such preventable risk factors. Although the risk factors associated with the development of CVDs are many and affect several processes in the body, there are two major underlying causes for CVD, namely hypertension and atherosclerosis [1]. Nutritional intervention is well accepted as a safe and effective approach to health maintenance and it has been estimated that a change in nutritional pattern may reduce cardiovascular-related deaths by 60% [4]. Seafood consumption has been linked to a reduced risk of these illnesses, and traditionally the beneficial effects have been associated with the long-chained omega 3 polyunsaturated fatty acids eicosapentaenoic acid (EPA, 20:5n3) and docosahexaenoic acid (DHA, 22:6n3) [5,6,7,8,9,10]. Emerging evidence has now demonstrated that the proteins, as well as other bioactive compounds, may also be relevant for improving human health by different mechanisms. Fish and seafood are excellent sources of good quality proteins that upon digestion may be sources for bioactive peptides with documented favorable physiological effects such as antioxidative, antihypertensive and other cardioprotective effects. The documentation is mainly from in vitro studies, but the number of preclinical studies and human trials performed is arising. This review aims to summarize these preclinical and clinical studies.

### Clinical Trials

In order to evaluate causal relationships between nutrients and chronic diseases, two main approaches are normally applied, namely epidemiological and experimental studies. There are advantages and disadvantages associated with both study types, and a combination of studies will probably return the most solid foundation for evidence. In brief, epidemiological studies range over a long period of time and include large population groups. Data material may be very large, there are few restrictions in diet and diseases can be included as endpoint. The main drawback is that they are poorly controlled and that the sources of error are many. Experimental studies are performed in a more controlled environment compared to epidemiological studies. Clinical trials involving human volunteers and preclinical trials involving animals fall into this category. In such studies, test subjects are enrolled into a controlled environment where their diets, together with other relevant measures, are regulated. Effects are registered through a range of different physiological parameters, depending on the aim of the study. 

In our opinion, experimental clinical studies on humans are by far the most accurate way to evaluate the health effects of different diets or food components. However, such studies are also very expensive, time consuming and complex. Further, inclusion criteria for participants may vary according to the aim of the study and comparisons between studies may therefore be difficult. 

## 2. Oxidative Stress and Antioxidative Status

Aerobic metabolism is accompanied by an inevitable production of reactive oxygen species, normally referred to as ROS. To reduce the production and counter the harm of these ROS, the human body is equipped with several antioxidant systems involving mechanisms that prevent free radicals from causing damage and mechanisms that repair or mitigate any occurred damage [11]. However, the balance between ROS and antioxidants may shift in favor of ROS, and a condition called oxidative stress arises. This condition has been related to several disorders, atherosclerosis [12] in particular. There is now a general acceptance that consumption of dietary antioxidants is an effective approach to increase the body’s antioxidant load and mitigate the effects of ROS [13]. The mechanisms may be inactivation of ROS [14], scavenging of free radicals [15], chelating of pro-oxidative transition metals [16] and reduction of hydroperoxides [17,18]. Some amino acids, in particular histidine, glutamic acid, aspartic acid, along with phosphorylated serine and threonine, have the ability to chelate prooxidative transition metals [16]. Usually, peptides are considered more potent antioxidants due to the stability of the resultant peptide radical [13]. The antioxidant potential of a protein or peptide depends on the amino acids being exposed and accessible to prooxidants. Increased exposure of amino acids can be attained by food processing, fermentation or gastrointestinal digestion. The in vitro antioxidant activity of marine protein hydrolysates has been shown for several fish species, mollusks, crustaceans and microalgae. The link to a beneficial health outcome in humans is, however, still on a theoretical level. Despite evidence showing clear associations between oxidative stress and CVDs, epidemiological data on antioxidant intake and disease prevention are inconclusive. Natural antioxidant intake from foods has been proven beneficial [19], whereas analyses with antioxidant supplementation have been proven unfavorable or even resulting in adverse effects in preventing all-cause mortality [20,21].

### 2.1. Human Studies

One study has been published focusing on the effect of marine proteins on oxidative stress and antioxidative status (Table 1). During a randomized parallel intervention, 276 overweight subjects were following a diet designed for weight loss [22]. The subjects were randomized to four groups and followed a diet plan with either lean meat, lean meat supplemented with omega-3 fatty acids, cod or salmon during eight weeks. The oxidation product malondialdehyd and the antioxidative capacity were measured before and after the trial. After the intervention period, the amount of the oxidation product was significantly reduced in the group following the cod based diet (from 1.81 nM to 1.72 nM). At the same time, the antioxidative capacity in this group increased significantly (from 0.62 nM to 0.71 nM) and was significantly higher than that in the individuals following both the lean meat diet and the lean meat with omega-3 capsules. It was suggested by the authors that the specific protein characteristics of cod or the high amount of taurine may have contributed to this effect.

### 2.2. Animal Studies

Documentation from preclinical trials has been increasing. Two studies investigated the effect of fish protein compared to casein in the feed for male spontaneously hypertensive rats (SHR) over a two months period. In one study, lipid peroxidation (measured as TBARS) in heart and liver were significantly lower in the SHR receiving the fish protein diet compared to the SHR receiving the casein diet [23], whereas no difference was observed in muscle and adipose tissue and higher lipid peroxidation was observed in kidney. The antioxidant status in heart and liver increased with the fish protein diet, whereas it remained unchanged in plasma during the feeding trial. This suggests that fish protein plays an important role in the antioxidative defense system in heart and liver, but not in plasma. In the second study 50%, of the SHR were induced with diabetes after one month, which resulted in increased plasma antioxidative status in the fish protein fed SHR compared to the casein fed SHR [24]. In a recent study by Jensen et al. [25], apolipoprotein E-deficient (apoeE−/−) mice were used to evaluate the effect of dietary cod and scallop on atherosclerotic burden and related parameters, among them gene expressions of antioxidative proteins. Twenty-four 5-week-old female apoeE−/− mice were fed Western type diets with chicken or cod and scallop as the protein sources for 13 weeks. It was shown that the hepatic endogenous antioxidant paraoxonase 2 (*Pon2* gene) was down regulated in mice fed the cod-scallop diet, suggesting lower oxidative stress in this group.

## 3. Atherosclerosis, Dyslipidemia and Inflammation

Atherosclerosis (originating from Greek: athero meaning gruel and sclerosis meaning hardness) is a complex, progressive and multifactorial inflammatory condition affecting the arteries. The arteries consist of three distinct layers: the outer layer, tunica adventitia, consists of flexible fibrous connective tissue, tunica media consists of smooth muscle cell tissue and elastic connective tissue, whereas the inner layer, tunica intima, consists of a membrane of collagen and glycoproteins lined by endothelial cells. The endothelial cells have a vast range of metabolic and regulatory functions, including transport of metabolic substances, regulation of vascular tone, defense against inflammation, angiogenesis and regulation of hemostasis and coagulation [26]. Disturbance of these regulatory processes, for instance by oxidative stress, is often the trigger for the onset of atherosclerosis. Under normal conditions, vasoactive substances are released from endothelial cells [27], but reduced bioavailability of these compounds, in combination with accumulated low density lipoprotein (LDL) could lead to activation of endothelial cells and subsequently a condition known as endothelial dysfunction [28]. Activation of endothelial cells leads to an inflammatory response involving the production of a cascade of chemokines, adhesion factors and integrins that are stimulated by transcription factors, such as nuclear factor kappa b (NFκB) [29]. These substances recruit monocytes to the endothelial surface, followed by adherence and transmigration into the intima. The influx of monocytes is often accompanied by influx of other inflammation cells, such as T-cells, dendritic cells and mast cells. Once placed in the intima, monocytes may differentiate into macrophages influenced by pro-inflammatory cytokines. Macrophages are phagocytic cells expressing scavenger receptors for uptake of modified LDL. The activated macrophages are programmed to protect our body against danger, and thus the normal processes for cholesterol handling and transport are impaired and accumulation of cholesteryl esters eventually leads to the formation of foam cells, and fatty streaks [30]. Continued inflammatory responses may further accelerate the atherosclerotic process. Stimulation of proliferation and migration of smooth muscle cells to the intima and release of intracellular contents (lipids, cholesterol) from both macrophages and smooth muscle cells, may build up a large plaque inside the intima. Protease secretion by macrophages degrade extracellular matrix, such as collagen, and a fibrous cap is formed around the excess lipids. Expression of collagen degrading enzymes can gradually weaken the fibrous cap leading to plaque rupture and release of intracellular content into the arteries, thrombus formation, and this may eventually result in myocardial infarction [31,32].

### 3.1. Inflammation

Very few studies documenting the effect of marine proteins on inflammation or parameters associated with inflammation are published (Table 1).

#### 3.1.1. Human Studies

The effect of lean fish on inflammatory gene expression has been investigated in two published studies, one study evaluated the effect of lean fish in patients with coronary heart disease [33] and another study evaluated the effect in healthy subjects [34]. In the study with coronary heart disease patients, 27 subjects were divided into three groups eating either lean fish or fatty fish four portions a week for eight weeks. One group served as control and did not consume fish during the intervention period. No effect on the inflammatory gene expression was observed in this study [33]. In the clinical trial with healthy individuals, 71 subjects were divided into five groups eating 400 g cod per week for eight weeks or the same amount of smoked salmon or fresh salmon. One group maintained their regular diet, and another group maintained their regular diet only supplemented with 15 mL cod liver oil. No changes in the measured inflammatory parameters were observed. Ouellet et al. [35] investigated the effect of cod protein compared to other animal protein sources on C-reactive protein. For four weeks, 19 insulin resistant, overweight subjects participated in a crossover study and were given a diet with 60% of proteins as cod or other animal sources. After the four weeks, the subjects returned to their normal diet for two weeks, before they switched to the opposite diet. C-reactive protein was reduced by 24% in the cod group compared to an increase of 13% in the group eating other animal protein sources. 

#### 3.1.2. Animal Studies

In a study published by Jensen et al. [25], apolipoprotein E-deficient (apoeE−/−) mice were also used to evaluate the effect of dietary cod and scallop on atherosclerotic burden and related inflammatory parameters. Twenty-two five-week-old female apoeE−/− mice were fed Western type diets with chicken or cod and scallop as the protein sources for 13 weeks. After the study period the mice given cod-scallop as the protein source had a 24% lower atherosclerotic plaque compared to the mice eating the chicken feed. Additionally, the cod-scallop group had a 19% lower expression of the inflammatory gene vascular cell adhesion molecule 1. Dort et al. [36] investigated the effect of cod on the resolution of inflammation in 128 male Wistar rats. For three weeks, the rats had free access to feed with cod protein or casein protein. Thereafter the leg was injured with bupivacaine. The results showed that the inflammation due to the bupivacaine injection resoluted earlier in the cod group. At 14 and 24 days post damage, the amount of neutrophile granulocytes was significantly lower in the cod group compared to the casein group. In another study by the same group, it was confirmed that the anti-inflammatory effect of cod was due to the amino acids arginine, glycine and taurine [37]. 

### 3.2. Dyslipidemia

Lipids such as cholesterol and triglycerides are highly hydrophobic and have to be transported by lipoproteins in the blood stream. Both LDL and high density lipoproteins (HDL) are important parts of the regulation of the cholesterol homeostasis in the body; LDL delivers cholesterol from the liver to the various organs, whereas HDL is important for the reverse transport from the organs back to the liver. An imbalance between these two lipoproteins in favor of LDL will lead to accumulation of cholesterol in the vasculature and in tissues other than the liver [38] and a condition, named dyslipidemia occurs [39]. This condition is one of the most prominent risk factors for the development of atherosclerosis. Elevated plasma concentrations of triglycerides have in several prospective studies been shown to make up a considerable risk factor for atherosclerosis [40,41]. Lowering of LDL cholesterol, by medicinal treatment or by lifestyle/dietary changes, has been adapted as a means to reduce the risk of atherosclerosis. Increasing the level of HDL cholesterol is considered as a way of reducing the risk of atherosclerosis. In addition to its reverse cholesterol transport properties, HDL is also associated with vasodilation [42].

#### 3.2.1. Human Studies

Several cross over studies have been conducted on healthy individuals comparing diets where lean seafood is the major protein source to diets with non-seafood, such as beef, chicken, eggs, and milk, as the major protein source (Table 1). A significant reduction in triglycerides was observed in some of the studies [43,44], whereas no significant difference between the diets was the conclusion in other studies [45,46]. In a study by Elvevoll et al. [47], 80 participants were given either a regular fish pate or a fish pate enriched with taurine. After the intervention period, subjects eating the taurine enriched fish pate experienced a reduction in cholesterol and LDL compared to the subjects eating fish pate without enrichment, suggesting an extensive beneficial effect of taurine. In other studies, the participants were selected based on being overweight, with a BMI over 27 (Table 1). In a double blind, randomized, placebo controlled study, 40 subjects were given supplements with fish protein or placebo over a period of eight weeks. No significant difference between the groups was observed for neither cholesterol, HDL nor triglycerides. LDL, however, was significantly reduced compared to baseline in the fish protein group [48]. Further the HDL/LDL ratio increased in the fish group during the intervention period. The effect of cod as the protein source in energy restricted diets for weight loss have been investigated in several studies [22,49,50]. In neither of the studies a significant effect on cholesterol was observed, whereas, in some studies, triglyceride levels were reduced compared to control diets [22,49]. In a crossover study by Ouellet et al. [35], cod was compared to other animal proteins in a four weeks crossover study with participants being overweight and diabetic (Table 1). In this study, the cholesterol and LDL was significantly reduced in the group eating the animal proteins compared to the group eating the cod protein. In addition, Erkkila et al. [51] conducted a clinical trial with patients with coronary heart disease (Table 1). For eight weeks the participants had either lean white fish or meat as protein source. No significant difference in blood lipids was found after the eight weeks. A similar study was repeated later with the same conclusion [52].

#### 3.2.2. Animal Studies

The effects of marine proteins on blood lipids have been investigated in both mice and rat models. Cod and scallop were compared to chicken and casein as protein sources to assess their effect on blood lipids in a high fat diet and in a Western diet [25,53]. The level of triglycerides was reduced after seven weeks on the high fat diet with cod and scallop, whereas no effects on total cholesterol or HDL-cholesterol were observed. In the other study with the Western diet, the mice eating cod–scallop had lower LDL-cholesterol compared to those eating chicken feed. Liaset et al. [54] divided 15 rats into three groups and fed them saithe hydrolysate, soy or casein as protein source for almost four weeks. The plasma concentration of triglycerides was reduced in the saithe hydrolysate fed rats compared to soy and casein fed rats. In two studies, spontaneously hypertensive rats were given feed with 20% fish protein compared to 20% casein. The group eating fish protein had significantly reduced total cholesterol in both studies [24,55]. In the latter study, the triglyceride levels were reduced after the intervention period as well. The combined effect of cod protein and oil on triglyceride metabolism has been investigated in rats [56]. The rats were fed different protein sources and oils during four weeks. Cod protein alone did not affect the level of triglycerides, whereas together with menhaden oil, the cod protein reduced triglyceride levels by 50% compared to casein.

### 3.3. Coronary Heart Disease

Coronary heart disease is a collective term for heart attack and angina pectoris. An epidemiological study evaluated the association between increased seafood consumption and reduced risk for coronary heart disease.

#### Human Studies

Bernstein et al. [57] followed 84,136 30–55 year old women in the Nurses’ health study (Table 1). The women in this study had no known cancer, diabetes mellitus, angina, myocardial infarction, stroke, or other vascular diseases, for 26 years. In a model which statistically controlled for energy intake, it was shown that one serving of fish per day was associated with a 30% reduction in risk for coronary heart disease compared with one serving of red meat.

## 4. Hypertension

The blood pressure is a measure of the heart’s ability to pump blood and is presented as systolic above diastolic pressure. Systolic designates the pressure of the pumping heart and diastolic designates the pressure of the relaxed heart. A blood pressure of 120/80 mmHg is regarded normal and if one or both numbers are elevated, the heart’s workload is increased and a condition called hypertension arises. This condition is one of the most important precursors for CVD, and is associated with heart failure, myocardial infarction and stroke [58], affecting almost one third of adults worldwide [59]. An increase of 20/10 mmHg above normal has been reported to double the risk of fatal CVD among people between 40 and 49 years [60].

The regulation of blood pressure is a complex process involving several mechanisms. Some of these, such as change of arteries diameter, regulation of blood volume in the blood stream and addition or removal of fluids in the blood stream, are purely mechanic, whereas others are more complex regulatory systems. One of these is the renin-angiotensin-aldosterone-system (RAAS). When blood flow or volume through the kidney decreases, the enzyme renin is excreted from the glomerulus. Renin cleaves angiotensinogen produced in the liver to form the decapeptide angiotensin I (Ang I). Angiotensin Converting Enzyme (ACE) produced mainly in the lungs further cleaves Ang I to the octapeptide Angiotensin II (Ang II) which constricts the arterial vessels and induces a rise of the blood pressure. In addition, it stimulates the adrenal cortex to produce aldosterone, which increases the reabsorption of sodium and water from the kidneys and further increases the blood pressure [61]. Another regulatory system is the kinin-kallicrein system (KKS). The ACE also participates in this system where it inactivates the vasodilator bradykinin [62]. Hence, inhibition of ACE will in both regulatory systems result in a prevention of blood pressure rising. In addition to being an independent risk factor for CVDs, high blood pressure is also recognized as a risk of atherosclerosis [63,64]. 

The effect of marine protein on blood pressure has been evaluated in several animal models, but limited data from epidemiological studies have suggested any association between fish intake and blood pressure.

### 4.1. Human Studies

The effect on blood pressure of lean fish as the protein source, has been evaluated and documented in two dietary intervention studies (Table 1). Erkkila et al. [51] randomized 33 medicated patients with coronary heart disease into three groups eating lean fish, fatty fish or lean meat as protein sources four times a week during eight weeks. After the intervention period, both systolic and diastolic blood pressure was reduced in the group eating lean fish. Ramel et al. [50] investigated the dose-response effect of number of cod meals per week. They randomized 126 healthy, overweight individuals into three groups, all following an energy restricted diet with either no cod, cod three times, or five times a week for eight weeks. The results from the blood pressure measurements were, however, inconsistent and therefore not reliable. Double-blind placebo controlled studies are generally regarded as a gold standard for evaluating the effect of different substances (Table 1). In one study, 34 overweight adults received supplementation of fish protein capsules or placebo tablets for eight weeks [48]. The intake of the supplement was 3 g per day the first four weeks and thereafter 6 g per day. No effect on blood pressure was observed. In another similar study, the effect of a salmon peptide on blood pressure was evaluated [65]. A number of 52 mild hypertensive individuals were divided into three groups drinking a beverage (50 mL/day) with 1 g, 0.3 g or no salmon peptide for four weeks. The systolic blood pressure was significantly reduced (140 to 135 mmHg) in the group receiving 1 g salmon peptide. Kawasaki et al. [66] evaluated the effect of a peptide administered to 29 individuals with high-normal blood pressure and mild essential hypertension. The subjects were randomized into two groups for a cross over placebo-controlled trial. The dipeptide drink significantly reduced the blood pressure in the dipeptide group, whereas no change was observed in the placebo group.

Results on blood pressure are not easily extrapolated between fish species, as taurine is known for its blood pressure reducing effect [67]. Taurine content varies greatly between fish species [68], however, compared to other foods, it is generally high in marine foods.

### 4.2. Animal Studies

While the documentation of blood pressure reducing effect in humans is scarce, several studies performed on animal are published. The, by far, commonest model for hypertension evaluation is using spontaneously hypertensive rats (SHR). These rats are bread to develop high blood pressure, and are well suited for monitoring through both acute and chronic studies. The majority of the studies published on the effect of marine protein on blood pressure, are acute studies. The SHR are given marine hydrolysates or peptides orally and the blood pressure has been measured before administration, just after administration up to several hours after administration. The first studies documenting the antihypertensive effect of bonito in SHR were published already in the 1990s [69,70,71]. Later, single oral doses of 10 mg/kg body weight of tuna hydrolysate [72,73] and yellow fin sole hydrolysate [74] have been shown to significantly reduce blood pressure. A blood pressure reducing effect has been documented in hydrolysates from shrimp [75], oyster [76], loach [77], sea cucumber [78], sardine [66], jellyfish [79], salmon [65,80], cobia [81] and skate [82]. In these studies, the test doses vary, making any comparison difficult. Nevertheless, the results may give an indication that marine protein in general is potential as a blood pressure reducing nutraceutical, food ingredient or food.

Some studies have also evaluated the chronic effect of marine hydrolysates on blood pressure. In such studies, the SHR are given the test items daily. Negative control is normally water or saline, while the positive control commonly is captopril. Jellyfish hydrolysate [79], sardine peptide [83] and sea bream hydrolysate [84] have all been tested in chronic studies. SHR have been administered daily over a period of four weeks with the hydrolysates in different dosages, resulting in significantly lowered blood pressure, even comparable to that of captopril. Hydrolysates of cod, haddock and salmon did not significantly reduce blood pressure in SHR during a four-week study [85], although the blood pressure in the group treated with cod hydrolysate did not increase after day 7. Fish has also been evaluated as part of the feed itself. Spontaneously hypertensive rats fed a standard chow supplemented with tuna hydrolysate, Katsuo-bushi, for seven weeks, experienced reduced blood pressure [71]. In three studies lasting for two months, SHR were fed standard animal chow where 20% of the feed was either fish protein or casein protein [24,55,86]. The blood pressure in the SHR eating fish protein was significantly reduced compared to that in those eating the casein protein. However, when this was investigated in rats with diabetes, no effect on blood pressure was observed [24].

## 5. Conclusions

Focus on health benefits from marine resources has traditionally been on the long chain omega-3 fatty acids. However, emerging evidence points out that other nutrients, such as peptides and proteins also play a major role. The current review sums up preclinical and clinical trials on the cardioprotective effects of marine protein and peptides. Clinical studies on humans are the superior method for evaluation of health effects, but also the most expensive, time consuming and complex way. The number of studies is thus quite low, but there are indications that marine proteins may have a positive effect on oxidative stress. Studies on inflammation parameters, blood lipid and hypertension are inconclusive. Further, as inclusion criteria for participants in each study vary greatly, depending on weight, gender, age and health status, conclusions from the different studies are difficult to draw and the clinical relevance is therefore limited. The number of animal studies published is larger and, particularly, the effects of marine proteins on hypertension are well documented. However, documentation of the effect on atherosclerosis and inflammation is scarce and further research on this field is also acquired. It is therefore of utmost importance to include more research from both animal, and most importantly, human studies on cardiovascular health effects of marine proteins and peptides.

## Figures and Tables

**Table 1:** Clinical trials investigating cardio protective of marine proteins and peptides.

Parameter	Study	Subjects, Inclusion Criteria	Protein Source	Result	Year	References
Oxidative stress	8 weeks, randomized parallel intervention	276 (4 groups), overweight, healthy	Cod, salmon, fish oil, control	Oxidation product reduced, AOC increased in cod group	2007	[22]
Blood pressure	8 weeks, double blind, randomized, controlled intervention	34 (2 groups), overweight	Fish protein capsules, placebo	No significant effect	2013	[48]
	8 weeks controlled, parallel dietary intervention	126 (3 groups), overweight	150 g cod 1/week, 150 g cod 3/week, no cod	No results	2009	[50]
	8 weeks controlled, parallel intervention	31 (3 groups), myocardial infarction	Lean fish, fatty fish, no fish	Blood pressure reduced in lean fish group	2008	[51]
	4 weeks double blind, placebo-controlled	52 (3 groups), mild hypertension	Salmon peptide, placebo	Systolic blood pressure reduced in peptide group	2008	[65]
	4 weeks randomized, double blind, placebo-controlled	29 (2 groups), high-normal blood pressure and mild essential hypertension	Sardine peptide	Blood pressure reduced in peptide group	2000	[66]
Inflammation	8 weeks, randomized, parallel dietary intervention	27 (3 groups) coronary heart disease	Lean fish, fatty fish, no fish	No significant effect	2009	[33]
	2 × 4 weeks crossover design	19 overweight/obesity insulin-resistance	Cod, other animal protein sources	24% reduction in plasma CRP	2008	[35]
Blood lipids	2 × 4 weeks, randomize, crossover design	20 healthy	Lean seafood, non-seafood	Reduced TG in lean seafood-group	2015	[43]
	4 weeks prospective, randomized crossover design	10 healthy	Lean seafood, beef diet	Reduced TG, cholesterol and VLDL	2009	[44]
	2 × 4 weeks, randomized crossover design	11 healthy men	Lean fish, non-fish	No significant effect	2000	[46]
	2 × 4 weeks crossover design	14 healthy premenopausal women	Fish, non-fish	No significant effect	1996	[45]
	7 weeks dietary intervention	80 (2 groups) healthy	Fish pate, fish pate with taurine	Reduced cholesterol and LDL with taurine	2008	[47]
	8 weeks, double blind, randomized, controlled intervention	34 (2 groups) overweight	Fish protein capsules, placebo	Reduced LDL in fish group compared to baseline	2013	[48]
	8 weeks, randomized parallel dietary intervention	276 (4 groups) overweight, healthy	Cod, salmon, fish oil, control	Reduced TG in cod-group	2007	[22]
	8 weeks, randomized, parallel dietary intervention	324 (4 groups), overweight	Lean fish, oily fish, control, fish oil	Reduced TG	2008	[49]
	8 weeks controlled, parallel dietary intervention	126 (3 groups) overweight	150 g cod 1/week, 150 g cod 3/week, no cod	No results		[50]
	2 × 4 weeks crossover design	19 overweight/obese insulin-resistant subjects	Cod, other animal protein sources	Reduced cholesterol and LDL	2008	[35]
	8 weeks controlled, parallel dietary intervention	31 (3 groups) subjects with myocardial infarction	Lean fish, fatty fish, no fish	No significant effect	2008, 2014	[51,52]
Coronary heart disease	Epidemiological study, 26 years	Healthy women aged 30–55	Fish	Reduced risk for coronary heart disease		[57]

AOC, antioxidative capacity; CRP, C-reactive protein; TG, triglycerides; VLDL, very low density lipoprotein; LDL, low density lipoprotein.
